# Massive glosso-cervical arteriovenous malformation: The rationale for a challenging surgical resection

**DOI:** 10.4317/jced.51608

**Published:** 2014-10-01

**Authors:** Raúl González-García, Isidoro Rubio-Correa, Carlos Moreno-García

**Affiliations:** 1 MD, PhD. Consultant Surgeon. Department of Oral and Maxillofacial-Head and Neck Surgery, Unversity Hospital Infanta Cristina, Badajoz, Spain; 2 MD. Resident Surgeon. Department of Oral and Maxillofacial-Head and Neck Surgery, Unversity Hospital Infanta Cristina, Badajoz, Spain; 3 MD. Consultant Surgeon. Department of Oral and Maxillofacial-Head and Neck Surgery, Unversity Hospital Infanta Cristina, Badajoz, Spain

## Abstract

Massive arterivenous malformations (AVM) in the cervico-facial area are rare but potentially life-threatening. Treatment protocols are not well-established. A 41-year old man presented large painless rubber-like mass within the entire neck, which also extended intraorally through the floor of the mouth, showing a slow growing pattern for 5 years. Angiography diagnosed it as cervicofacial AVM. Treatment approach consisted on the embolization of the right upper thyroid, lingual and facial arteries under intravenous sedation. Three days later, bilateral radical neck dissection and subtotal glossectomy was performed. A musculo-cutaneous pectoralis major pedicled flap was harvested to reconstruct the floor of the mouth. Treatment of massive AVMs in the cervico-facial area is challenging due to the associated disfigurement and frequent recurrence rate due to incomplete resection. Also, massive bleeding may be present despite pre-operative super-selective embolization. A new case is presented with focus on surgical treatment considerations.

** Key words:**Arteriovenous malformation, high-flow vascular malformation, cervical region, tongue, surgical resection

## Introduction

Vascular malformations are collections of abnormal vessels displaying a flat non-proliferative endothelium that most frequently develop in the adulthood, although are always present at birth and grow progressively (1). They can be classified in low-flow [capillary, venous, lymphatic] and high-flow vascular malformations [typically arteriovenous malformations – AVMs]. AVMs may accurately activate following trauma, infection, puberty, pregnancy or iatrogenia (2). Intra-operative bleeding is one of the most hazardous complications in the surgical management of high-flow vascular malformations. It is even more relevant for massive AVM within the cervical region, where the presence of vital vascular structures, such as the carotid artery and jugular vein may evolve in uncontrollable bleeding. Despite this associated high surgical risk, the presence of severe symptoms and signs, such as dyspnea, dysphagia, disfigurement and massive bleeding with subsequent life-threatening upper airway compromise makes surgery mandatory. The most appropriated management is based upon careful embolization followed by total resection, within the first 48-72 hours (3). Unfortunately, this goal cannot always be assumed without an associated intolerable morbidity, while the incomplete resection of the AVM may shortly evolve in recurrence due to the reorientation in blood supply and altered hemodynamics.

The case presented here clearly illustrates the aggressiveness of a giant AVM involving the whole anterolateral cervical region bilaterally from the infrahyoid muscles up to the mobile tongue, and also gives some recommendations for a safer complete surgical resection.

## Case Report

**Figure 1:**
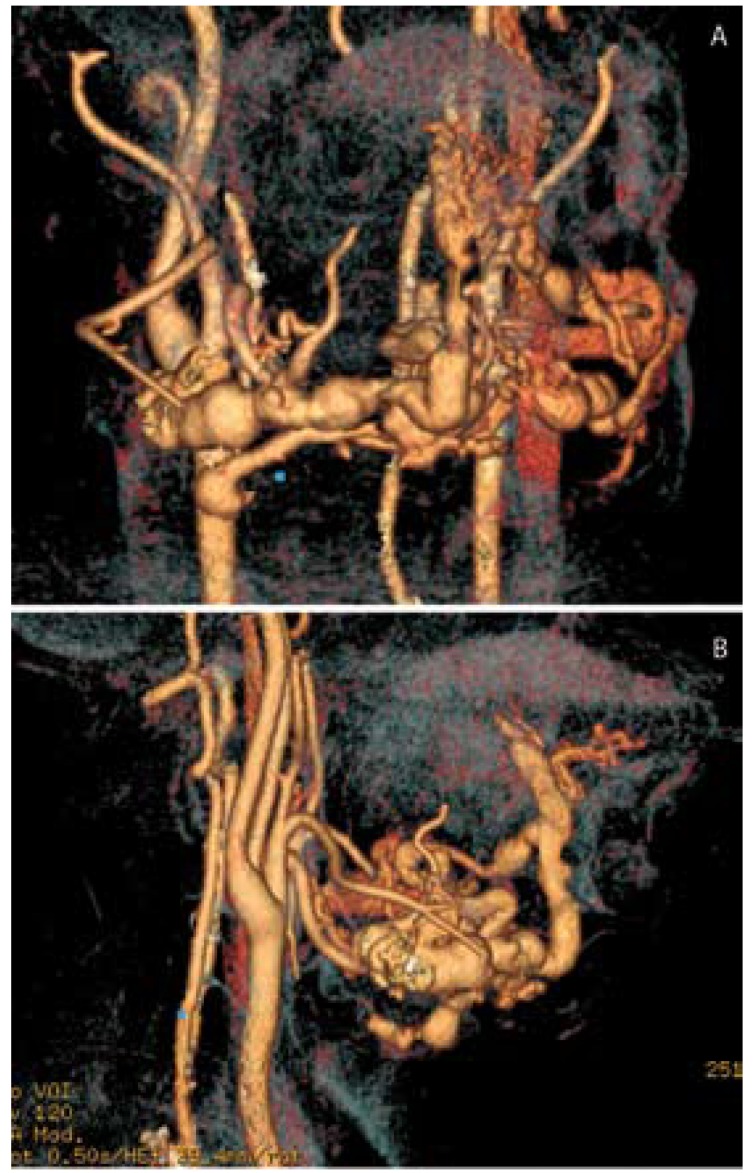
Angiography of both carotid arteries showing the AVM. A. Frontal view; B. Rigth Lateral view.

A 41-year old man presented with a large cervical mass involving both sides of the neck, with associated macroglossia for 5 years. No personal history of disease was reported. Physical examination revealed a large painless rubber-like mass within the entire cervical region, which also extended intraorally through the floor of the mouth, pushing it upwards. The tongue was largely involved with lingual mandibular imprints and also marked protrusion and displacement of the teeth over the vestibular region both in the upper maxilla and mandible. Progressive limitation of breathing, swallowing and speech was observed. A cervicofacial CT-scan showed a large mass in the floor of the mouth with involvement of the tongue, mylohyoid and geniohyoid muscles and anterior cervical soft tissues, formed by multiple tortuous small and large-size vessels, with preservation of the hyoid bone and both submaxillary glands. No pathologic lymph neck nodes were observed. Angiography of both external carotid arteries confirmed the vascular nature of the mass, this particular case being an arteriovenous malformation [AVM] mainly supported by the right lingual artery, but also by the facial and upper thyroid arteries. The mass presented large draining veins crossing the midline that finished at the left jugular vein (Fig. 1).

**Figure 2:**
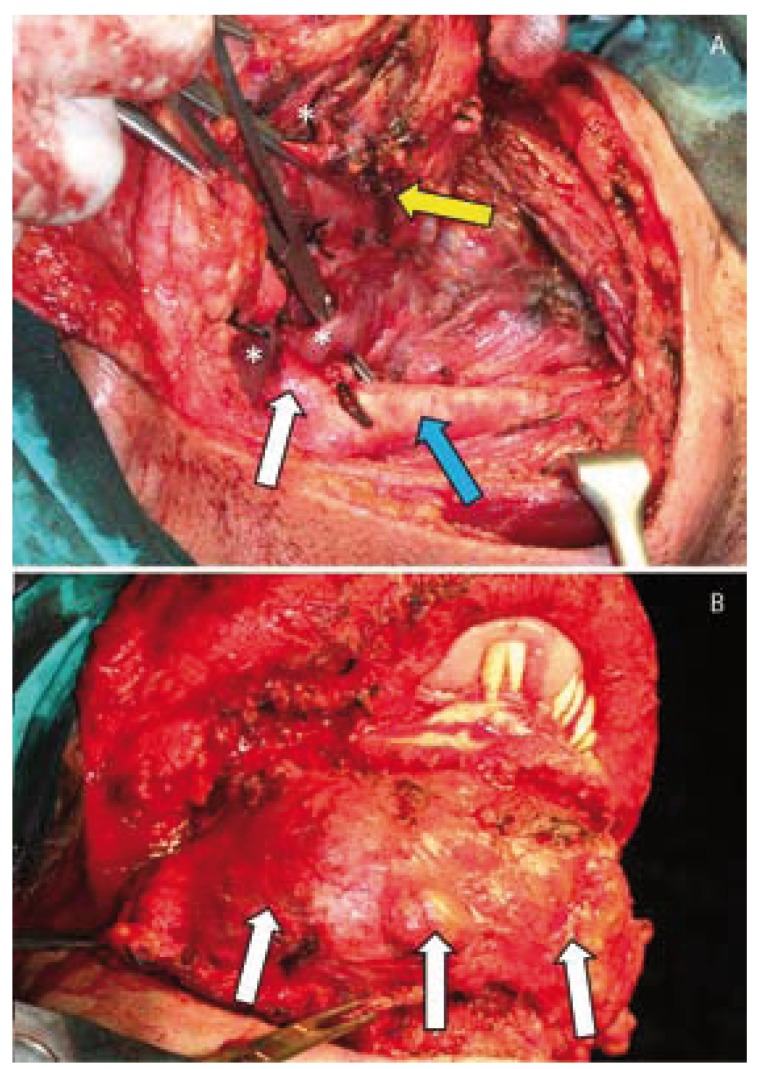
A. Intra-operative view, showing dissection of the AVM inferiorly (yellow arrow). Note the common carotid artery dissected (blue arrow) as well as the external carotid artery (white arrow). Large vessels of the AVM are signaled by asterisks; B. Intra-operative view, showing the entire mass in the cervical area (white arrows) partially dissected, as well as the exposure of the mandible after elevating a visor flap for a mandibular degloving. Note protrusion of the involved tongue.

**Figure 3:**
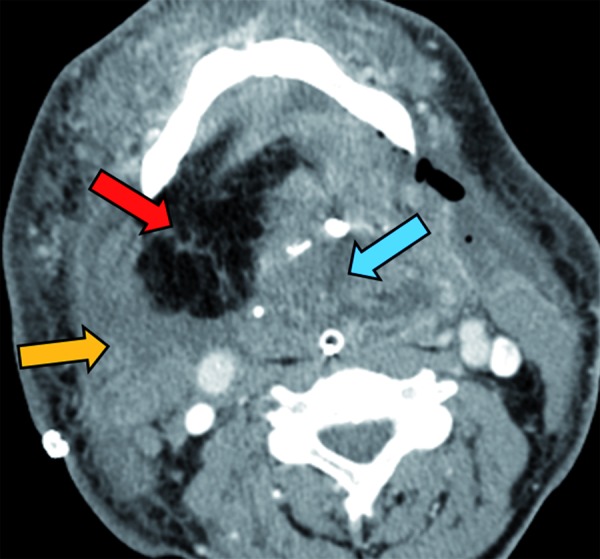
Post-operative CT-scan. Reconstruction of the resected floor of the mouth and bilateral cervical radical dissection with a musculocutaneous pedicled pectoralis major flap. Note the muscular component of the flap (orange arrow) and the fat tissue component (red arrow) covering the entire floor of the mouth. Remnant 1/3 posterior tongue is signaled with a blue arrow.

Treatment approach consisted on the embolization of the right upper thyroid, lingual and facial arteries under intravenous sedation followed by surgical resection 3 days later. Embolization was tried with rounded beads [microspheres] of polymers and collagen. Unfortunately, embolization was scarcely successful, as it was checked during open surgery. Tracheostomy was performed under local anaesthesia. Under general anaesthesia and intubation by the tracheostome, a bilateral cervicotomy form the right to left mastoid apophyses was performed to gain access to all the cervical levels. A visor flap was elevated for a facial degloving, followed by a median mandibulotomy and mandibular swing. This approach allowed wide access to the entire mass communicating the neck and the oral cavity. Both internal and external carotid arteries and jugular veins were exposed. The right upper thyroid, lingual and facial arteries were dissected and transected at their exit from the external carotid artery. This maneuver allowed a safer dissection of the mass both through a lateral and inferior approach. The thyroid gland was preserved, while resection was performed until the pre-laryngeal, infrahyoid and suprahyoid muscles were exposed. Further dissection along this layer was performed up to the submental region (Fig. 2). An V-Y resection of the anterior two thirds of the tongue together with the mylohioid, genihyioid and genioglossus muscles was performed with the aid of the harmonic blade to better control bleeding. In bloc resection of the entire specimen was completed with the harmonic blade (Fig. 2). A musculo-cutaneous pectoralis major pedicled flap was harvested to reconstruct the floor of the mouth (Fig. 3).

## Discussion

Failure of conservative treatment has been reported in the management of high-flow vascular malformations (3). This condition makes radical surgery imperative, although massive bleeding may occur intraoperatively. Noteworthy the risk of lethal bleeding or the sacrifice of vital structures have to be pre-operatively considered for this type of AVM, and the patient may be completely informed about the possibility for the occurrence of potential life-threatening complications, among them ischemic brain injury, massive uncontrollable bleeding and compressive haematoma.

**Figure 4:**
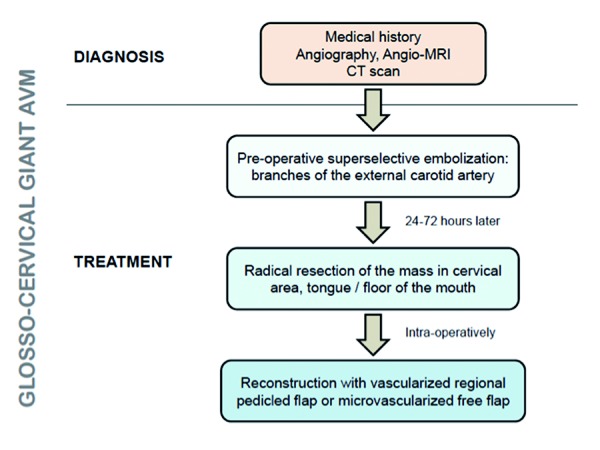
Scheme of a rationale approach for the treatment of glosso-cervical giant arteriovenous malformation (AVM).

Since AVMs are rare in the head and neck, limited experience is available concerning the surgical treatment of cervicofacial massive AVMs. The rationale for an adequate management of these lesions must be clearly established pre-operatively (Fig. 4), while adequate support by the anesthesiologist with enough blood supplies must be present in the event of a massive or maintained intra-operative haemorrhage.

Jackson *et al*. (3) reported the compartmentalization technique by the use of large, curved needles and non-absorbable sutures to interrupt the blood supply from the regional vascular network, after which high doses of the sclerosant agent tetradecyl sulfate 3% was injected into each compartment. The authors reported less bleeding while perfoming the ulterior surgical resection. Embolization alone is not a valid alternative for most massive AVMs, as it will result in the reorientation of blood supply and recurrence. Equally, partial resection of the AVM usually leads to re-expansion of the lesion 1 to 2 years post-operatively (4,5).

This is why resective surgery is advisable for most of cervico-facial AVMs. While most authors recommend surgery within the first 48-72 hours post-embolization, others (6) suggest that resection should be performed from 5 days to 6 weeks after embolization, because only in cases of effective embolization re-expansion does not occur within the first 6 weeks. Pre-operative embolization has been tried to decrease intraperative bleeding and provide a dry operative field. Several authors (2) have obtained good results with the use of polyvinyl alcohol foam [PVA] or acrylic microspheres, although embolization has the potential to cause stroke, bleeding, skin necrosis, blindness, pulmonary embolism or cranial nerve ischaemia (1). Unfortunately, the present case did not benefit from pre-operative super-selective embolization with microspheres, as intra-operative bleeding was not substantially reduced. It seems that pre-operative embolization in such large masses may not be as effective as in other less expansive lesions, as a result of multiple high-flow feeding vessels.

Surgery of massive giant AVMs, such as the one presented here, must be focused on entire resection of the specimen and adjacent involved soft tissues. Koshima *et al*. (6) also noted that extensive wide and deep surgical excision was a key point to prevent re-expansion of cervicofacial AVMs, over a long period of time. Whenever the mandible or the upper maxilla was affected, in block resection of bone by segmental mandibulectomy or maxillectomy should be performed to avoid persistent disease and local bone destruction. Extension of the cervical AVM up to the tongue with infiltration of the floor of the mouth and tongue may require extensive approaches in order to facilitate a wide exposure of the specimen to be excised. In bilateral cases, as it was ours, the first maneouvre was directed to the control of the arterial branches feeding the mass, at the exit of both external carotid arteries. Once tied, a lateral-inferior approach may allow a safer resection of the specimen, while dissecting the lesion deeply in a layer superficial to the infra- and suprahioid muscles. By a mandibular swing, dissection of the specimen is progressed to include the entire floor of the mouth and tongue. The harmonic blade is especially useful to achieve adequate haemostatics and to provide a safer separation of the mass form draining venous vessels, although feeding arterial vessels have to be secured with double non-absorbable ties.

As presented here, reconstruction should be performed to cover the generated defect in the cervical region and also the floor of the mouth. A pectoralis major myocutaneous flap is bulky enough to cover the excised cervical region, to protect the cervical vascular axis, and to provide a reliable support for the remnant tongue. Indeed, the use of myocutaneous flap reconstruction has been reported to prevent the recurrence of AVMs (2). Yamamoto *et al*. (7) when using free skin grafts to cover the generated defects, observed that changes in the vascularity and fibrosis of the wound contributed further to the ischemic environment, with subsequent recurrence of the AVM. Tark & Chung (8) reported that the use of vascularized free flaps may block the vicious cycle of ischemia. However, the selection of recipient vessels may be a problem due to potential arterial obstruction near the lesion secondary to pre-operative embolization (6). Regarding this, regional myocutaneous pedicled flaps, such as the pectoralis major, may obviate the necessity of a microvascular anastomosis, while providing good vascularization and enough bulk to cover the neck and reconstruct the excised floor of the mouth. Extensive defects involving the lips, cheeks and other facial soft tissues may require reconstruction with distant free flaps.

In summary, surgeons must be aware of the severity of this type of AVMs in terms of pre-operative and intra-operative life-threatening conditions. For massive cervico-facial AVMs, bleeding is not the exception, but the rule, as pre-operative embolization is frequently unsuccessful. Whenever achievable, reconstruction with vascularized pedicled or free flaps must be performed to cover the defect or the surgical field.
